# Angina at Low heart rate And Risk of imminent Myocardial infarction (the ALARM study): a prospective, observational proof-of-concept study

**DOI:** 10.1186/s12872-015-0140-z

**Published:** 2015-11-14

**Authors:** Yuk-ki Wong, Shelley Stearn, Sally Moore, Beverley Hale

**Affiliations:** Department of Cardiology, St. Richards Hospital, Western Sussex Hospitals NHS Foundation Trust, Spitalfield Lane, Chichester, West Sussex PO19 4SE UK; Statistics and Research, University of Chichester, Chichester, UK

**Keywords:** Unstable angina, Myocardial infarction, Myocardial preinfarction syndrome, Acute coronary syndrome, Heart rate

## Abstract

**Background:**

Myocardial infarction (MI) is often preceded by unstable angina. Helping patients identify the onset of unstable angina rather than MI may result in earlier treatment and improve outcomes. Unstable angina is angina occurring at a lower-than-usual workload. Since heart rate (HR) is correlated with degree of exertion, we hypothesised that angina occurring at low HR is a warning signal for unstable angina and MI.

**Methods:**

In this prospective study, 111 patients with acute coronary syndrome (ACS) or prognostically significant coronary disease were recruited. Each patient’s HR was measured using a portable electrocardiogram (ECG) recorder after regular class III exercise on the Canadian Cardiovascular Society Angina Grading Scale and the cumulative moving average and three-sigma (standard deviation) range were calculated for each new measurement. The HR was subsequently measured at the beginning of angina; a HR lower than the preceding three-sigma ranges for class III or anginal HR was regarded as a ‘warning signal’. The proportion of warning signals associated with ACS occurring in the following 2 weeks was compared with that for non-warning signals.

**Results:**

Nine cases of ACS occurred in eight patients. Two cases were preceded by warning signals; a signal marked the onset of ACS in a third patient, and four patients failed to make anginal ECG recordings. There were 591 documented episodes of angina during the study and ECGs were available for 383 (64.8 %) of these of which 55 were warning signals. Of these warning signals, 4 occurred in the 2 weeks preceding ACS, compared with 4 of 328 non-warning signals (odds ratio, 6.4; 95 % confidence interval, 1.5–26.2; *p* = 0.01; positive predictive value, 7.3 %; negative predictive value, 98.8 %).

**Conclusions:**

Low HR angina may identify unstable angina and serve as an early warning for MI. In addition, angina that does not occur at a low heart rate indicates that ACS is very unlikely.

## Background

Myocardial infarction (MI) should ideally be treated in the first ‘golden hour’. However, most patients do not seek help until 2 h after symptom onset [1, 2] and treatment delays can occur after hospital arrival [3]. Although some patients are taught to recognise the onset of a heart attack [4, 5], ideally patients should seek intervention prior to occurrence of the critical ischaemic event. In theory, this should be possible because more than 50 % of heart attacks are preceded by a prodromal period (up to 2 weeks or more) of unstable angina, in which angina occurs on reduced exertion [6–10]. Helping patients to identify the onset of unstable angina rather than MI may result in the ability to provide earlier treatment and improve outcomes.

Heart rate (HR) has a linear relationship with cardiac output [11] and is used as a measure of exertion during treadmill exercise testing, during which angina occurring at a low HR is considered to be more clinically significant than that occurring at a high HR [12]. Therefore, we hypothesised that if patients could measure their HR at the onset of angina and if an episode occurred where the HR was significantly lower than that of previous episodes, this might be a warning indicator of unstable angina. Since many patients with diagnosed coronary disease are asymptomatic due to therapy, they may have no previous anginal episodes with which to compare the HR. However, because the general consensus is that asymptomatic patients who subsequently experience angina of at least class III severity on the Canadian Cardiovascular Society Angina Grading Scale may be at high risk for MI [13], a reasonable comparator HR under these circumstances is one corresponding to exertion normally associated with class III angina; i.e., one flight of stairs or ‘one to two blocks’ on a flat surface at a normal pace [14]. We therefore investigated whether angina occurring at a low HR is a marker of unstable angina and MI.

## Methods

### Study overview

All participants gave informed consent and ethical approval was obtained from Brighton West Research Ethics Committee (Ref No 09/H1111/23). The study was included in the National Institute for Health Research Clinical Research Portfolio (study ID 6974) and the study period was July 2009 to October 2011.

### Inclusion and exclusion criteria

Patients aged 54 years or older at relatively high risk for subsequent acute coronary syndrome (ACS) were recruited. Two groups were included in the study. The first group was enrolled within 6 weeks of hospital admission and comprised patients with ACS characterised by angina of at least 5-min duration associated with either dynamic electrocardiogram (ECG) changes or an elevated serum troponin I level. The second group had significant coronary disease diagnosed by coronary angiography (stenosis of at least 70 % in the left main artery or proximal left anterior descending artery and one other coronary artery or in all three main coronary arteries) but revascularisation had either been declined or was considered to be impossible or inappropriate. The main exclusion criteria were a pacemaker in situ and chronic arrhythmia.

### Study protocol and ECG measurements

All patients were given a pocket ECG recorder and instructed to carry it with them at all times (MD100B; Beijing Choice Electronic Technology Co., Ltd. or PC-80B; Shenzhen Creative Industry Co., Ltd.). A label displaying advice to seek help in case of angina lasting ≥15 min despite the use of sublingual glyceryl trinitrate every 5 min was positioned to hide the displayed HR.

During an initial consultation, the patients were observed using the ECG recorder before and after a 300 m walk and before and after a climb of one flight of stairs (12 steps of 17 cm height), equivalent to class III exertion. The patients were asked to identify a walk of similar distance near their home, including stairs where available, and the HR was measured before and after these activities daily for 7 days and weekly thereafter. The patients made recordings as soon as possible after the start of angina and documented the details in a diary. The quality of documentation and recordings were checked at 2 weeks and, where necessary, at 4 weeks. If quality was poor after 4 weeks, the patients were excluded; otherwise, they had follow-up visits every 3 months. At each visit, supervised class III exercises were performed and ECGs recorded using the patient’s ECG recorder. All exercises were preceded by 15 min of rest.

There were no restrictions on treatment; however, if medication changes were expected to affect HR or a revascularisation procedure or ACS occurred, unsupervised class III exercises were performed daily for the 7 days following the therapy change before reverting to a weekly schedule.

### ECG analysis

ECG data were downloaded at each visit and manually analysed. For each 30 s recording, the HRs corresponding to the first and last five consecutive R waves were calculated; ectopic beats were allowed if the first and last R wave of any run was a sinus beat. The difference in HR between the first and last run was used as an estimate of the speed of HR decline following exercise. If there were no five-beat runs, four- or three-beat runs were used, and the relative proportion of these runs was used as a measure of the quality of ECG recordings. If no three-beat runs could be identified, the ECG was considered uninterpretable. Additional measures of ECG quality were how early the first run started and how late the second run started. The HR from the first run was used when different ECGs were compared.

### Outcome measures

The main outcomes were unstable angina and MI occurring after enrolment. MI was diagnosed according to the universal definition [15]. Unstable angina was diagnosed if angina occurred at rest or persisted for more than 15 min after MI had been excluded. Atypical angina was defined as symptoms that patients thought were angina but that differed from previous anginal symptoms. The cause of death was ascertained from the death certificate.

### Statistical analysis

All statistical analyses were performed using IBM SPSS Statistics for Windows, Version 21.0 (IBM Corp., Armonk, NY). A two-tailed 0.05 significance level was used for all tests. For each patient, the cumulative moving average and standard deviation (SD) for angina and class III HRs were recalculated with each new measurement, but following changes in chronotropic medications or revascularisation and ACS, readings prior to these events were disregarded. Also for each patient, the coefficients of variation (CV) were calculated for class III HRs after 7 days and at 3, 6, 12, and 18 months following enrolment to assess the variation of HR measurements over time. For each episode of angina, the HR was compared with the three-sigma (SD) ranges for preceding angina and preceding unsupervised class III exercises. If the HR was below any of these ranges, angina was considered to have occurred at a low HR and represented a ‘warning signal’. Otherwise, it was a ‘non-warning signal’. The proportion of warning signals preceding a diagnosis of ACS in the subsequent 2 weeks was compared with that for non-warning signals using the *χ*^2^ test with calculation of the odds ratio (OR), 95 % confidence interval (CI), positive predictive value (PPV), and negative predictive value (NPV).

## Results

In total, 111 patients were recruited for this study, and their compliance with unsupervised class III exercises was good (≥74 %) (Table 1). For angina, 43 patients reported this symptom with 591 documented episodes and ECGs were available for 383 (64.8 %) of these of which 55 were signals and 328 were non-warning signals. For all indications, there were 29,593 ECGs and 94.5 % of these were interpretable (Table 1).

**Table 1:** Patient characteristics, ECG findings, and clinical events

Patient characteristics	
Number of patients recruited	111 (13 withdrew)
Number of patients not recruited due to poor quality of ECG recordings or diary documentation	4
Male	87 (78.4 %)
Mean age (SD)	68.1 years (8.0)
Recruited with prognostically significant coronary disease found at cardiac catheterisation	38 (34.2 %)
Recruited after acute coronary syndrome	73 (65.8 %)
Mean follow-up period between first and last heart rate recording (SD)	445.6 days (204.6)
Prescribed a negative chronotropic drug at start of study	92 (82.9 %)
Mean resting supine heart rate at start of study (SD)	58.4 bpm (9.4)
Number of patients with ECG recordings	
Unsupervised 300-m walk	111 (100 %)
Unsupervised flight of stairs	82 (73.9 %)
Angina	43 (38.7 %)
Number of diary-documented events	
Unsupervised 300-m walk (percentage of expected)	6705 (85.4 %)
Unsupervised flight of stairs (percentage of expected)	4082 (74.2 %)
Angina	591
Interpretable ECGs for diary-documented events	
Unsupervised 300-m walk^a^	5144 (76.7 %)
Unsupervised flight of stairs^a^	3423 (83.9 %)
Angina	383 (64.8 %)
Supervised 300-m walk^a^	688
Supervised flight of stairs^a^	685
ECG quality (interpretable ECGs)	
ECGs with two heart rate measurements	96.5 %
Heart rate calculations based on five consecutive R waves	93.4 %
Mean start of first heart rate calculation (SD)	2.0 s (3.8)
Mean start of second heart rate calculation (SD)	25.2 s (4.2)
Mean difference in heart rate between the beginning and end of 30-s ECGs	
Rest ECG for unsupervised 300-m walk (SD)	−0.8 bpm (4.0)
Postexertional unsupervised stairs (SD)	−2.0 bpm (5.7)
Postexertional unsupervised 300-m walk (SD)	−4.1 bpm (5.0)
Angina (SD)	−2.0 bpm (5.4)
Clinical events during follow-up	
ST elevation myocardial infarction	0
Non-ST elevation myocardial infarction (number of patients)	2 (2)
Unstable angina (number of patients)	6 (5)
Coronary death diagnosed postmortem	1

^a^ECGs were recorded at rest and post-exercise, but the numbers refer to interpretable post-exercise ECGs

**Fig. 1:**
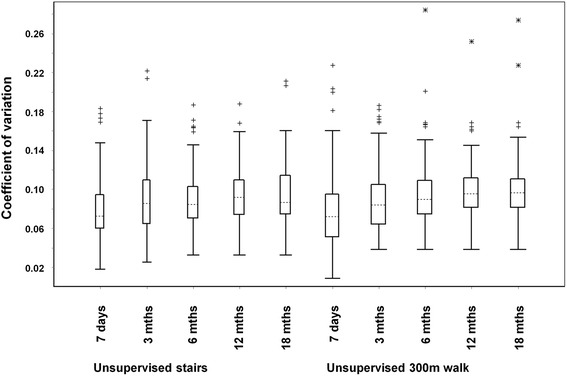
Box-and-whisker plots for coefficients of variation of heart rate measurements after various time periods

The CVs for HR measurements tended to increase with time (Fig. 1), and class III exercise CVs were lowest after the daily readings from the first 7 days.

During follow-up, nine cases of ACS occurred in eight patients (Table 1). Four cases of ACS were characterised by a single episode of severe and prolonged pain for which no ECG was recorded. In a fifth case, the patient missed a clinic visit due to feeling unwell and died 1 week later without an ECG recording. This patient did not have prior heart failure and the post-mortem diagnosis was ‘left ventricular failure secondary to coronary disease’. In a sixth case, the patient’s unstable angina was caused by unexplained nocturnal sinus tachycardia. A seventh case had a warning signal at the onset of unstable angina (rest pain) whilst the eighth case was preceded by a warning signal that occurred 1 day prior to further pain and a diagnosis of Non-ST elevation MI. The ninth case was preceded by 4 warning signals of which the earliest occurred 68 days prior to diagnosis of unstable angina with another 6 signals occurring after diagnosis.

In terms of warning signals, 4 of 55 occurred during the 2-week period preceding ACS compared with 4 of 328 non-warning signals (OR, 6.4; 95 % CI, 1.5–26.2; *p* = 0.01; PPV, 7.3 %; NPV, 98.8). In total, 20 patients had at least one signal, but 6 patients had symptoms that were judged to be atypical, accounting for 45.5 % of all signals. When patients with atypical chest pain were excluded from the analysis, the corresponding proportions were 4 of 30 compared with 4 of 253 (OR, 9.6; 95 % CI, 2.3–40.6; *p* = 0.002; PPV, 13.3 %; NPV, 98.4 %).

**Fig. 2:**
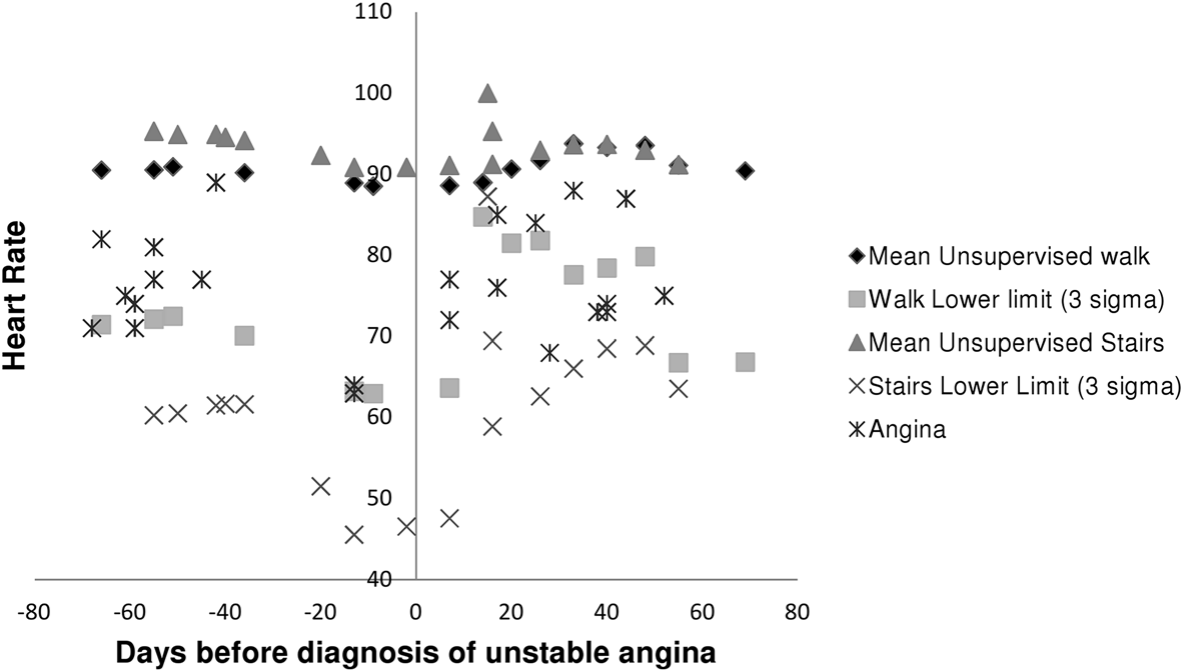
Heart rates before and after diagnosis of unstable angina for the ninth case of ACS

For the patient who had 4 signals prior to diagnosis of unstable angina, there was a prodrome of angina occurring on less exertion. During this period, there was a gradual reduction in comparator HRs and widening of the corresponding three-sigma ranges, which was more marked when climbing stairs (Fig. 2). Anginal HRs were also lower during this period; however, because of the wider three-sigma ranges for the comparator HRs, a signal was only generated when anginal HRs were compared with the walk, and not with the stairs or preceding angina. In total, 10 patients had signals along with comparator HRs from both stairs and walking. Of their 42 signals, only 6 were triggered by both comparators.

Two additional patients had signals associated with histories strongly suggestive of ACS but they did not seek medical attention at the time and a diagnosis of ACS could not be definitely confirmed.

## Discussion

We found that episodes of angina occurring at a low HR in patients with known coronary disease were more likely to occur in the 2 week period leading up to an ACS. The case for accurately identifying prodromal symptoms in order that ‘myocardial infarction might be averted in many instances’ has been made before [8]. To the best of our knowledge, however, the present study is the first to use HR to objectively identify unstable angina. Another important finding of this study is that angina which does not occur at a low heart rate indicates that a diagnosis of ACS is very unlikely with a NPV of 98.8 %. This compares with a PPV of 13.3 % at best. Unfortunately, angina HR data was missing from 4 cases of ACS. In addition, 2 patients with warning signals did not present at the time of their likely ACS. These missing data may have adversely affected the PPV. The clinical implications of these findings are that warning signals may help patients identify unstable angina and non-warning signals may help to reduce the significant number of patients admitted to hospital with chest pain but who do not have ACS.

We defined a ‘warning signal’ HR for angina as one that was less than the lower limit of comparator three sigma ranges (i.e., a 1:370 chance that the warning signal had occurred by chance); choosing this level of stringency minimised the possibility of false positive results. The sensitivity for detecting a signal HR is increased if the CVs for the comparator HRs are small, since the associated three sigma ranges will be narrower. In this study, the Class III comparator three sigma ranges were continuously re-calculated and over time, the CVs tended to increase (Fig. 1). It was found that the initial seven daily measurements were sufficient to achieve the lowest CV and an alternative strategy would be to recalculate the three sigma ranges with seven new daily measurements at regular intervals such as after every 3 months.

We found that during the prodrome of unstable angina, there can be widening of the comparator three sigma ranges that made it difficult to detect a signal (Fig. 2). It has been reported that most cases of unstable angina are less than 2 weeks in duration. Therefore, a possible algorithm could be to use a comparator period ending at least 2 weeks before the episode of angina under consideration.

We found in one patient that signals may continue in the period after ACS has been diagnosed. We speculate that such signals may indicate persisting clinical instability. From a diagnostic point of view, such signals cannot be considered an early warning since they have occurred after the event. However, in our pre-specified analysis, we did not distinguish between signals that occurred before or after the event but in future studies and for the purposes of making a more accurate assessment of predictive values, it may be best to exclude all signals and non-warning signals after an ACS until patients are clinically stable. Similarly, in the same patient, we found that signals could precede the clinical diagnosis by up to 68 days. In this study, signals occurring earlier than 2 weeks before diagnosis were false positives but in this patient, it could be argued on clinical grounds that they were true positives. Nevertheless, it has been reported in the literature that most cases of unstable angina last for up to 2 weeks and that is why we chose this as a cut-off for an early warning period.

A high proportion of signals were associated with symptoms that were considered to be atypical for angina. To reduce such false positive signals, patients could be advised that the character of angina rarely changes, except in diabetics and after cardiac surgery.

### Strengths and limitations of this study

The main strengths of this study are its prospective design and the lack of restrictions on treatment. In fact, prescription of negatively chronotropic drugs was high (Table 1) and yet, signal HRs were recorded from 20 of 43 patients who had angina.

A limitation of our study was the relatively small sample size because despite the fact that we had selected a high risk population, there was a smaller than anticipated number of ACS events. It remains to be seen whether our findings would be replicated in a population with lower risk coronary disease although there appears to be no biological reason to expect a difference. Less than 50 % of heart attacks occur de novo in patients without known coronary disease and an interesting question would be how successful a strategy of measuring HR would be in people presenting for the first time with chest pain that could be angina.

Unfortunately, HR data were only available for 64.8 % of documented angina episodes and patients did not always have their ECG recorders with them. It is possible that modern technology which increases the portability and availability of suitable devices may improve the compliance for HR measurements.

### Comparison with other studies

We are not aware of any other study that has attempted to determine whether an episode of angina is unstable by measuring a physiological variable. In clinical practice, patients are taught to recognise the features of chest pain that may indicate the onset of MI [5] but still, patients often present late. Even in this study where patients received continuous reminders of the characteristics of unstable angina, 2 patients failed to seek medical attention at the time of unstable symptoms. Further investigation is needed to determine whether HR data indicative of unstable angina coupled with an appropriate alarm helps patients to seek medical attention earlier. Hopefully, patient self-monitoring will augment the traditional approach of patient education and improve clinical outcomes.

## Conclusions

Low HR angina may identify unstable angina and warn of impending myocardial infarction. In contrast, angina at higher heart rates indicates that ACS is very unlikely. Therefore, HR data may help high risk patients receive earlier treatment whereas it may reduce hospital admissions amongst those without ACS. Whether anginal HR can improve clinical outcomes needs to be tested in an interventional study.
